# Alamandine/MrgD axis prevents TGF-β1-mediated fibroblast activation via regulation of aerobic glycolysis and mitophagy

**DOI:** 10.1186/s12967-022-03837-2

**Published:** 2023-01-13

**Authors:** Wei Wang, Yue Zhang, Wenhui Huang, Yafei Yuan, Qiaohui Hong, Zhanzhan Xie, Lijuan Li, Yixin Chen, Xu Li, Ying Meng

**Affiliations:** 1 Department of Respiratory and Critical Care Medicine, Nanfang Hospital, Southern Medical University, Guangzhou, 510000 China; 2 Department of Emergency Medicine, Nanfang Hospital, Southern Medical University, Guangzhou, 510000 China; 3 Ministry of Education, Key Laboratory of Hainan Trauma and Disaster Rescue, College of Emergency and Trauma, Hainan Medical University, Haikou, 571199 China

**Keywords:** Pulmonary fibrosis, Fibroblast activation, Alamandine/MrgD axis, Mitophagy, Aerobic glycolysis

## Abstract

**Background:**

Idiopathic pulmonary fibrosis is a chronic progressive, lethal disease in which ectopic lung fibroblast (LF) activation plays a vital part. We have previously shown that alamandine (ALA) exerts anti-fibrosis effects via the MAS-related G-protein coupled receptor D (MrgD). Here, we further investigate how it moderates transforming growth factor β1 (TGF-β1)-induced LF activation by regulating glucose metabolism and mitochondria autophagy (mitophagy).

**Methods:**

In vitro, we examined glycolysis-related protein hexokinase 2 (HK2), 6-phosphofructo-2-kinase/fructose-2,6-biphosphatase 3 (PFKFB3), and lactic acid in cells treated with TGF-β1. The oxygen consumption rate and the extracellular acidification rate were detected using Seahorse assays. Then, mitophagy was evaluated using transmission electron microscopy, mt-Keima, and the co-localization of Parkin and COX IV with LC3 and LAMP1, respectively. The autophagic degradation of HK2 and PFKFB3 was detected by 3MA and bafilomycin A1 and assessed by their co-localization with LC3 and LAMP1, respectively. The effects of ALA on LF activation markers collagen I and α-SMA were detected. The effects of ALA on glucose metabolism, mitophagy, and the activation of LF were also investigated in vivo.

**Results:**

We found that the ALA/MrgD axis improved TGF-β1-mediated LF activation by repressing glycolysis by downregulating HK2 and PFKFB3 expression. Lactic acid sustained positive feedback between glycolysis and LF activation by maintaining the expression of HK2 and PFKFB3. We also showed that glycolysis enhancement resulted from blocking the autophagic degradation of HK2 and PFKFB3 while upregulated mRNA levels by TGF-β1, while all of those improved by ALA adding. Importantly, we determined that moderation of Parkin/LC3-mediated mitophagy by TGF-β1 also promotes glycolysis but is reversed by ALA. Furthermore, we proved that ALA counteracts the effects of bleomycin on HK2, PFKFB3, LC3, Parkin, and LF activation in vivo.

**Conclusion:**

In this study, we show that the ALA/MrgD axis prevents TGF-β1-mediated fibroblast activation via regulation of aerobic glycolysis and mitophagy.

**Supplementary Information:**

The online version contains supplementary material available at 10.1186/s12967-022-03837-2.

## Introduction

Aerobic glycolysis has a crucial role in the development of idiopathic pulmonary fibrosis (IPF) and transforming growth factor β1 (TGF-β1)-induced lung fibroblast (LF) activation [1, 2]. Targeting hexokinase 2 (HK2), the first key enzyme in the process of glycolysis, or 6-phosphofructo-2-kinase/fructose-2,6-biphosphatase 3 (PFKFB3), which produces fructose-2,6-bisphosphate, the most potent allosteric activator of the glycolytic rate-limiting enzyme phosphofructokinase-1, can alleviate TGF-β1-induced LF activation and lung fibrosis [3, 4]. However, the specific molecular mechanisms of the activation and degradation of HK2 and PFKFB3 by TGF-β1 remain obscure.

Mitophagy is a type of selective autophagy that clears damaged and potentially cytotoxic mitochondria through the autophagosome-lysosome pathway to maintain cellular homeostasis [5, 6]. As demonstrated by our previous research and that of others, mitophagy has an important but undefined role in multiple experimental animal models and human lung diseases and is associated with lung dysfunction and disease progression [7–9]. Mitophagy-deficient mice are more vulnerable to developing lung fibrosis [10]. Mitochondria represent the powerhouse and center of metabolism in eukaryotic cells, and evidence indicates that mitophagy is closely associated with metabolic disorders [11]. However, it is unclear whether mitophagy regulates key glycolysis enzymes HK2 and PFKFB3. The autophagosome–lysosome pathway is one of the main mechanisms of intracellular protein degradation [12]. There is evidence that HK2 and PFKFB3 may be degraded by this pathway, but whether they affect LF activation in IPF is unknown [13, 14].

Furthermore, it has been reported that simultaneous inhibition of autophagy and glycolysis can inhibit tumor growth, suggesting a close complementary relationship between glycolysis and autophagy [15]. Although it is tempting to postulate a single driver mediating LF activation and IPF, this is unlikely, given the multifactorial nature of pro-fibrotic events. Here, we focus on HK2 and PFKFB3 to better understand the mechanisms involved.

Alamandine (ALA), a novel member of the renin–angiotensin–aldosterone system (RAAS), exerts anti-inflammation and anti-fibrosis effects via the ALA/MrgD axis [16, 17]. Our previous studies have demonstrated the anti-fibrosis and autophagy-modulating effects of ALA in the lungs and liver [18, 19]. However, further studies are needed to investigate whether ALA protects against LF activation by regulating Parkin/LC3-mediated mitophagy. Moreover, different components of RAAS have been shown to be closely related to metabolic disturbance, but whether ALA plays a part in LF activation by improving glucose metabolism is unclear [20]. Building on previous findings, we performed experiments to explore the specific mechanism of how ALA regulates fibroblast activation and PF via mitophagy and aerobic glycolysis.

## Materials and methods

### Reagents

ALA was purchased from Biosyntan (Berlin, Germany); D-Pro7-Ang-(1–7) and β-alanine were synthesized by Sangon (Shanghai, China); TGF-β1, 3PO, 2DG, and bafilomycin A1 were purchased from MedChemExpress (HY-100558, USA); 3-methyladenine (3MA) was purchased from Selleck (S2767, USA); and Mdivi-1 was purchased from Abmole (M2830, USA). MrgD siRNA and Parkin siRNA were provided by Ribobio (Guangzhou, China). L-lactic acid was purchased from Solarbio (IL0540, Beijing, China). Other reagents were as described below.

### Animal experiments

Male wild-type C57BL/6 mice (6–8 weeks old) were purchased from the Central Animal Care Facility of Southern Medical University, and the experiments were approved by the Committee on the Ethics of Animal Experiments of Southern Medical University and conducted following the regulations of the institution (Permit No. NFYY 2022-0153). Mice were housed in a specific-pathogen-free room with 12-h light/dark cycles and controlled temperature and humidity, with free access to water and food. Mice were randomly divided into three groups (10 mice per group): a control group, bleomycin (BLM) treatment group, and BLM + ALA treatment group. Initially, 100 μL of sterile saline containing BLM at a single dose of 5 mg/kg was instilled into the tracheas of mice in the two BLM groups after the mice were anesthetized using 1% pentobarbital (30 mg/kg); an equivalent dose of sterile saline was administered to the control group. ALA was continuously infused into the BLM + ALA mice using a subcutaneously implanted micro-osmotic pump at a rate of 2 μg/kg/h for 28 days. The other two groups simultaneously received the same dose of saline as a control. Animals were euthanized 28 days after establishing the PF model. The mice were anesthetized using pentobarbital before sample collection. Lung tissues were removed and fixed in 4% paraformaldehyde or stored at − 80 °C for further histological and biochemical analyses.

### Cell isolation and culture

Primary LFs were isolated from the lungs of C57BL/6 male mice as previously described [21]. The cells were cultured in Dulbecco’s modified Eagle medium (DMEM; C11995500BT-1, GIBCO, USA) containing 15% fetal bovine serum (FBS; FSP500, GIBCO, USA) plus 1% Pen-Strep (10,000 U/mL penicillin and 10,000 U/mL streptomycin; 15140122, GIBCO, USA) at 37 °C in a 5% CO_2_ incubator.

### Isolation of mitochondria

Fibroblast mitochondria were isolated using a mitochondrial isolation kit (C3601, Beyotime, China) according to the manufacturer’s instructions. Briefly, cells were dispersed and centrifuged to obtain cell pellets. Then, they were resuspended with 1 mL lysis buffer and transferred into a glass grinder for homogenization. The homogenate was subsequently centrifuged at 800*g* for 5 min at 4 °C. The supernatant was collected and then centrifuged at 15,000*g* for 10 min at 4 °C, followed by giving up the medium buffer. Finally, 60 μL of lysis buffer containing protease inhibitor was used to resuspend the pellet to obtain the mitochondrial protein.

### L-lactic acid and pH adjustment

L-lactic acid (IL0540, Solarbio) at 10 mM concentration was added to DMEM containing 15% FBS. Then, the pH of the medium was adjusted to 7.6 using 1 mol/L NaOH before it was used for the incubation of cell cultures.

### Expression plasmid construction and transfection

An expression plasmid containing PFKFB3 (accession: NM_001177756) was generated by the following steps. Briefly, PFKFB3 cDNA reverse transcribed from mRNA (extracted from primary LFs) was amplified using PCR. The PCR amplicon appended with sites for restriction digest (BamHI and XhoI) was inserted into the pEnCMV plasmid (P27576, MiaoLing, Hubei, China). The resulting PFKFB3 plasmid was sequenced by Sangon Biotech (Shanghai, China). Transfection was performed using Lipofectamine 3000 (L3000015, ThermoFisher Scientific, USA) according to the manufacturer’s protocol. The efficiency of transfection was assessed by western blotting and quantitative reverse transcription PCR (qRT-PCR). The primary LFs were subjected to subsequent experiments after 24 h of transfection.

### siRNA transfection

The siRNA transfection in fibroblasts was performed using Lipofectamine 3000 (L3000015, ThermoFisher Scientific) according to the manufacturer’s protocol.

### Measurement of oxygen consumption rate and extracellular acidification rate

Oxygen consumption rate (OCR) and extracellular acidification rate (ECAR) were determined using a Seahorse XFe96 Extracellular Flux Analyzer (Agilent, CA, USA). Briefly, cells were seeded in XF96 Cell Culture Microplates and incubated at 37 °C for 24 h. The culture medium was changed to XF assay medium supplemented with 1 mM pyruvate, 2 mM glutamine, and 10 mM glucose for OCR assay or 1 mM glutamine for ECAR assay, and cells in the new medium were placed in a 37 °C incubator without CO_2_ for 1 h. OCR was measured by sequential injections of 1 µM oligomycin, 0.5 µM carbonyl cyanide 4-(trifluoromethoxy) phenylhydrazone, and 1 µM rotenone plus antimycin A to perform a mitochondrial stress test using the XF Extracellular Flux Analyzer. ECAR was examined under basal conditions and during the sequential injection of 10 mM glucose, 1 µM oligomycin, and 50 mM 2-DG. After each assay, cells were lysed, and the protein concentration was measured to normalize the OCR and ECAR.

### Extracellular lactate analysis

Primary mouse LFs were pretreated with 100 nM ALA for 1 h and then exposed to 4 ng/mL TGF-β1 (HY-P7117, MedChemExpress, USA) for 24 h. Then, the supernatant was collected to measure extracellular lactate production (A019-2-1, Nanjing Jiancheng, China) according to the manufacturer’s instructions. Lactate levels were measured at 530 nm using a microplate reader.

### Mitotracker and lysotracker staining

Mitochondria were examined using Mitotracker Green (C1048, Beyotime, China), and lysosomes were examined using Lysotracker Red (C1046, Beyotime, China). Primary LFs were incubated for 20 min at 37 °C in DMEM supplemented with 20 nM Mitotracker Green and 50 nM Lysotracker together and protected from light. After washing with phosphate-buffered saline three times, cells were photographed under a confocal microscope (LSM880, Carl Zeiss, Germany).

### Measurement of mitophagy using mt-Keima

Cells were transfected with the pH-dependent fluorescent protein mitochondria-targeted monomeric Keima-Red (mt-Keima, Genechem, Shanghai, China), a protein that emits green light (ex485 nm) at a neutral pH and emits red light in acidic lysosomes (ex561 nm). Then, the cells were pretreated with or without ALA for 1 h, followed by TGF-β1. Twenty-four hours later, live cells were observed with a confocal microscope (LSM 980, ZEISS, Germany). Then, using ImageJ software, ten cells per group were chosen randomly, and the mitophagy ratio was calculated as the ratio between the red area and the green area.

### Mitochondrial superoxide detection

Mitochondrial superoxide levels were detected by MitoSOX™ Red (M36008, Invitrogen, USA) according to the manufacturer’s instructions. Briefly, live cells were incubated with MitoSOX Red (5 μM) for 10 min at 37 °C in the dark. Following washing with warm HBSS/Ca/Mg, cells were imaged by confocal microscopy.

### Histopathological and immunohistochemical analyses

For histological analysis, lung tissue sections were subjected to hematoxylin and eosin (H&E) and Masson staining, and the severity of lung fibrosis was analyzed by Ashcroft scoring. For immunohistochemical staining, paraffin-embedded mouse lung sections were incubated with primary antibodies against PFKFB3 (1:200, A5593, Bimake, USA), HK2 (1:200, 66974-1-Ig, Proteintech, USA), and α-SMA (1:200, 19245T, Cell Signaling Technology, USA) according to the manufacturer’s instructions (GK500705, DAKO, Denmark). Images were captured by microscopy (IX73, Olympus or Imager D2, Carl Zeiss).

### Immunofluorescence staining

Paraffin sections (4 µm) and cells were prepared for immunofluorescence and incubated with primary antibody overnight at 4 °C. Then, lung sections and cells were stained with the appropriate secondary antibody and mounted with DAPI (F6057, Sigma Aldrich, USA). Images were captured with a confocal microscope (LSM880, Carl Zeiss) or fluorescence microscope (BX63, OLYMPUS). The primary antibodies used included anti-PFKFB3 (1:200, A5593, Bimake), anti-HK2 (1:100, 66974-1-Ig, Proteintech), anti-Parkin (1:100, 66674-1-Ig, Proteintech), anti-cytochrome c oxidase IV (COX IV; 1:100, 66110-1-Ig, Proteintech), anti-LC3B (1:100, A5202, Bimake/1:200, 83506S, Cell Signaling Technology), anti-LAMP1 (1:100, 32731, Signalway Antibody, USA), and anti-Collagen I (1:100, ab260043, Abcam, USA/1:100, 67288-1-Ig, Proteintech).

### Western blot analysis

Equal amounts of proteins from cell lysate or tissues, as determined by BCA protein estimation, were boiled with 5× loading buffer and separated by 10% or 12% sodium dodecyl sulfate–polyacrylamide gel electrophoresis. The proteins were transferred to 0.22 μm polyvinylidene fluoride membranes and then blocked with 5% bovine serum albumin for 1 h at room temperature. Next, the membranes were incubated with primary antibodies, including antibodies against collagen I (1:1000, 14695-1-AP, Proteintech), α-SMA (1:1000, 19245 T, Cell Signaling Technology), PFKFB3 (1:1000, A5593, Bimake), HK2 (1:1000, 66974-1-Ig, Proteintech), β-actin (1:1000, AM1021B, Abcepta, China), LC3B (1:1000, A5202, Proteintech), VDAC1 (1:1000, A5224, Bimake), and Parkin (1:1000, 66674-1-Ig, Proteintech) overnight at 4 °C. The membranes were washed three times with TBS-T buffer and then incubated with secondary antibodies (FDR007, FDM007, Fdbio Science, China) at room temperature for another 1 h. After further washing of the membranes three times with TBS-T buffer, protein signals were detected using a BLT GelView 6000 Pro system.

### qRT-PCR

RNA was extracted using TRIzol (9109, Takara, Japan), and cDNA was synthesized using PrimeScript™ RT Master Mix (RR036A, Takara, Japan). qPCR was performed using TB Green™ Premix Ex Taq™ (RR420B, Takara, Japan) with a Roche LightCycler^®^ 480. The primers used in this study are presented in Table 1. The relative fold change was calculated by the comparative CT method.

**Table 1:** Oligonucleotide primer sequences for qRT-PCR

Gene	Primer direction	Sequence
*Pfkfb3*	Forward	CGGGAGAGGTCAGAGAACATGAA
	Reverse	CCTCCATTCTCCCGAGTCCA
*Hk2*	Forward	GCCTCGGTTTCTCTATTTGGC
	Reverse	TGGTAGAGATACTGGTCAACCTTC
*Acta2*	Forward	AGGGCTGTTTTCCCATCCATCG
	Reverse	TCTCTTGCTCTGGGCTTCATCC
*Col1a1*	Forward	GGAGGGCGAGTGCTGTGCTTT
	Reverse	GGGACCAGGAGGACCAGGAAGT
*Tubulin α1*	Forward	GGATGCTGCCAATAACTATGCTCGT
	Reverse	GCCAAAGCTGTGGAAAACCAAGAAG

### RNA-seq data analysis

The RNA-seq data of lung tissue and fibroblasts isolated from IPF patients were downloaded from GEO DataSets of NCBI (GEO accession: GSE185492, GSE52463). Heatmap analysis was performed using the pheatmap (v1.0.12) package via R scripts.

### Statistical analysis

All the results are expressed as the mean ± SD. Data analysis was performed using GraphPad Prism 8. Intergroup comparisons of mean values were performed by one-way analysis of variance. Statistical significance was defined as *P* < 0.05.

## Results

### ALA/MrgD axis attenuated bleomycin-induced pulmonary fibrosis and TGF-β1-induced fibroblast activation

**Fig. 1:**
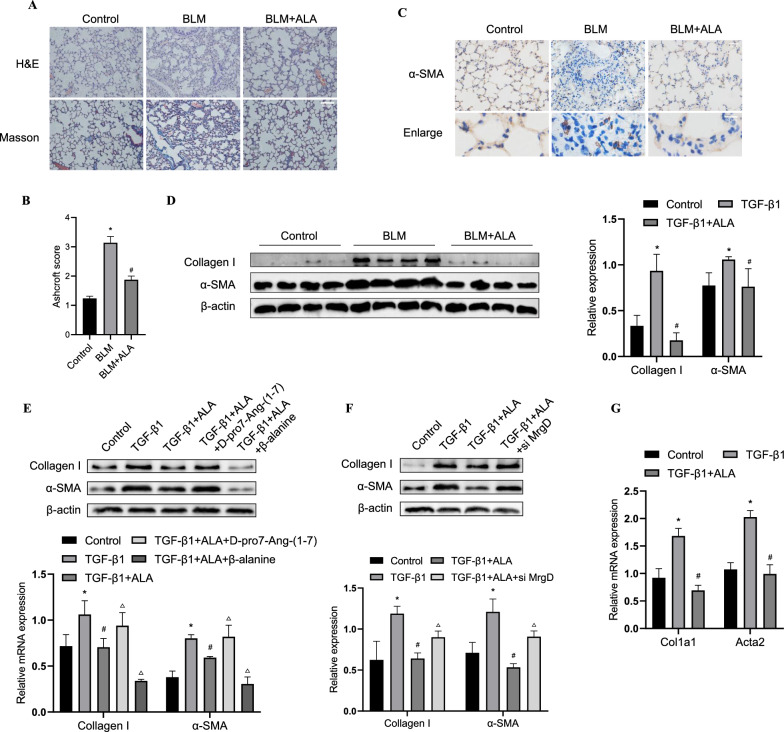
ALA attenuates bleomycin-induced lung fibrosis and TGF-β1-induced lung fibroblast activation. **A** Representative images of lung sections stained with H&E and Masson’s trichrome. Bar: 100 μm. **B** Ashcroft scores for each group. **C** Immunohistochemical staining was performed to determine the localization and expression of α-SMA. Bar: 50 μm. **D** Protein levels of collagen I and α-SMA in lung tissues were analyzed by western blotting. **E**, **F** Protein levels of collagen I and α-SMA. **G** mRNA levels of *Col1a1* and *Acta2* in fibroblasts. Original magnification: 200×. Data are presented as mean ± SEM. *P < 0.05 versus vehicle group; ^*#*^P < 0.05 versus BLM group or TGFβ1 group; ^△^P < 0.05 versus the TGFβ1 + ALA group (n = 3)

To explore the precise mechanism of pulmonary fibrosis, BLM-induced lung fibrosis was established. Sections of lung tissue from the BLM group displayed severe lung fibrosis compared with the control group as determined by H&E and Masson staining and Ashcroft score (Fig. 1A, B). The expression of α-SMA in lung tissue also increased in the BLM group, as detected by immunohistochemistry (Fig. 1C). Furthermore, the protein expression of collagen I and α-SMA was enhanced in BLM-induced fibrotic lung (Fig. 1D). By contrast, the BLM + ALA group showed an apparent reduction in lung fibrosis (Fig. 1A, B). Moreover, treatment with ALA inhibited the expression of collagen I and α-SMA in the BLM + ALA group (Fig. 1C, D). In vitro, the protein and mRNA levels of fibroblast markers collagen I and α-SMA in primary LFs were significantly decreased in the TGF-β1 + ALA group compared with the TGF-β1 group, and the MrgD receptor agonist β-alanine enhanced the effect of ALA whereas the MrgD receptor antagonist D-pro7-Ang-(1-7) or siRNA knockdown of MrgD had the opposite effect (Fig. 1E–G). These data suggest that the ALA/MrgD axis has an attenuating effect on TGF-β1-induced fibroblast activation and BLM-induced PF.

### ALA/MrgD axis inhibited fibroblast metabolic reprogramming induced by TGF-β1

**Fig. 2:**
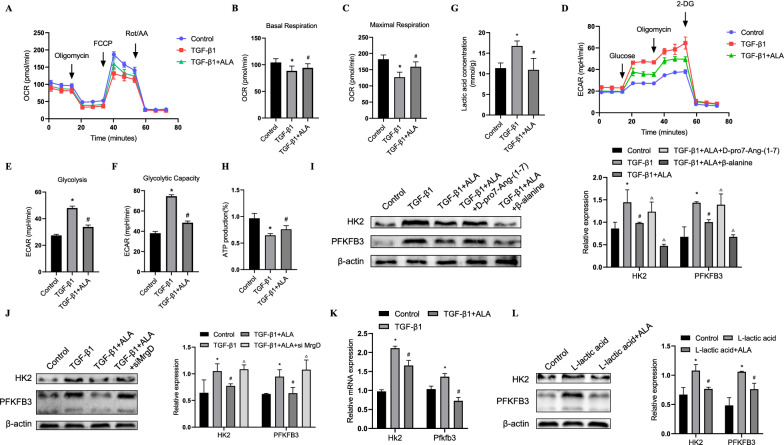
ALA/MrgD axis inhibited glycolysis in TGF-β1-induced activated fibroblasts. **A** OCR was measured using the Seahorse XF. Basal respiration (**B**) and maximal respiration (**C**) are quantified and shown as histograms. **D** ECAR was measured using Seahorse XF. Glycolysis (**E**) and glycolysis capacity (**F**) are quantified and shown as histograms. Extracellular lactate concentration (**G**) and intracellular ATP levels (**H**). Protein (**I**, **J**, **L**) and mRNA (**K**) levels of PFKFB3 and HK2 in fibroblasts. Data are presented as mean ± SEM. *P < 0.05 versus control group; ^#^P < 0.05 versus TGF-β1 group or L-lactic acid group; ^△^P < 0.05 versus TGFβ1 + ALA group (n = 3)

Next, we investigated whether the ALA/MrgD axis inhibited TGF-β1-induced metabolic reprogramming, and some glycolysis indicators were tested. We found that ALA treatment increased OCR levels compared to the TGF-β1 group (Fig. 2A–C). In addition, ECAR analysis showed that TGF-β1 treatment increased glycolysis and glycolytic activity, both of which were reduced by ALA (Fig. 2D–F). Furthermore, ALA significantly decreased extracellular lactic acid levels but increased ATP production compared with the TGF-β1 group (Fig. 2F, G), indicating that metabolic reprogramming induced by TGF-β1 was mitigated by the ALA/MrgD axis. Next, to determine the precise mechanism by which ALA regulates glucose metabolism, we detected the expression of PFKFB3 and HK2, two key enzymes of the glycolytic pathway. TGF-β1 upregulated their expression at both the protein and mRNA levels, whereas these effects were reversed by the ALA/MrgD axis (Fig. 2I–K). As lactic acid could induce LF activation [22] and ALA could downregulate lactic acid (Fig. 2G), we next explored whether lactic acid could induce glycolysis, with ALA addition having the opposite effect. We found that L-lactic acid alone upregulated the expression of HK2 and PFKFB3, whereas ALA reversed this effect (Fig. 2L). These data suggest that the induction of glycolysis by TGF-β1 could be counteracted by the ALA/MrgD axis through multiple targets.

### ALA inhibited HK2 and PFKFB3 expression in bleomycin-induced pulmonary fibrosis

**Fig. 3:**
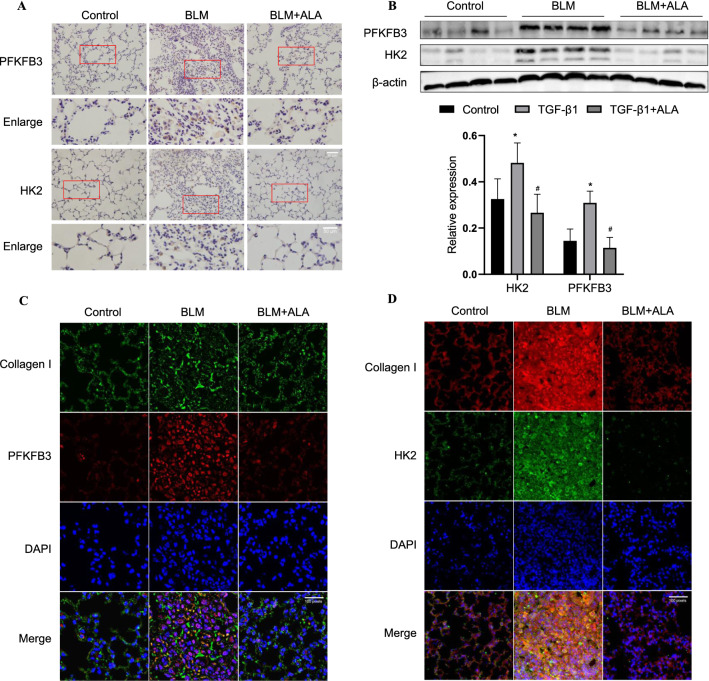
ALA inhibited activation of glycolysis in bleomycin-induced pulmonary fibrosis. **A** Immunohistochemical staining to determine the localization and expression of HK2 and PFKFB3. Bar: 50 μm. **B** Protein levels of PFKFB3 and HK2 in lung tissues. **C**, **D** Co-localization of PFKFB3 and HK2 with collagen I in lung tissues. Original magnification: 200× or 100 pixels. Data are presented as mean ± SEM. *P < 0.05 versus vehicle group; ^#^P < 0.05 versus BLM group

Next, we tested whether ALA could inhibit the expression of HK2 and PFKFB3 in vivo. The expression of PFKFB3 and HK2 was increased by BLM but decreased by ALA infusion (Fig. 3A). The protein levels in lung tissue displayed the same tendency (Fig. 3B). Then, using collagen I as a marker of fibroblasts, we found that the co-localization of collagen I and PFKFB3 or HK2 was elevated in the BLM group but decreased by ALA (Fig. 3C, D). These findings reveal that HK2/PFKFB3-mediated glycolysis in fibroblasts has a non-negligible role in BLM-induced lung fibrosis.

### ALA inhibited fibroblast activation by repressing glycolysis

**Fig. 4:**
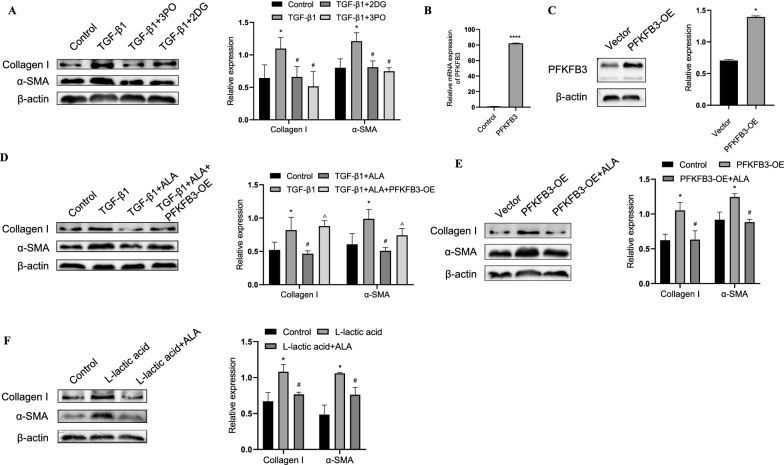
ALA inhibited lung fibroblast activation by repressing glycolysis. **A** Protein levels of collagen I and α-SMA in fibroblasts treated with TGF-β1 with or without 3PO and 2DG, the inhibitor of PFKFB3 and HK2, respectively. **B**, **C** mRNA and protein expression of PFKFB3 in fibroblasts processed with PFKFB3 overexpression plasmid or mock plasmid. **D**–**F** Collagen I and α-SMA expression. Data are presented as mean ± SEM. *P < 0.05 versus control group; ^#^P < 0.05 versus TGF-β1 group or PFKFB3 overexpression group or L-lactic acid group; ^△^P < 0.05 versus TGFβ1 + ALA group (n = 3)

Then, we explored whether ALA exerted an unfavorable effect on fibroblast activation by repressing glycolysis. 3PO and 2DG, inhibitors repressing PFKFB3 and HK2, respectively, could counteract the pro-fibrotic effect of TGF-β1 (Fig. 4A), which is in line with previous research [4, 23]. Next, we constructed a PFKFB3 overexpression plasmid and transferred it to fibroblasts (Fig. 4B, C). PFKFB3 overexpression reversed the effect of ALA on TGF-β1-induced fibroblast activation (Fig. 4D). Overexpression of PFKFB3 alone promoted the expression of collagen I and α-SMA in fibroblasts, and this phenomenon was repressed by ALA (Fig. 4E). L-lactic acid alone also induced fibroblast activation, but this was repressed by ALA (Fig. 4F). Taken together, the above data demonstrate that ALA inhibits fibroblast activation by repressing TGF-β1-induced PFKFB3/HK2/lactate-mediated glycolysis.

### ALA increased Parkin/LC3B-driven mitophagy in fibroblasts and fibrotic lungs

**Fig. 5:**
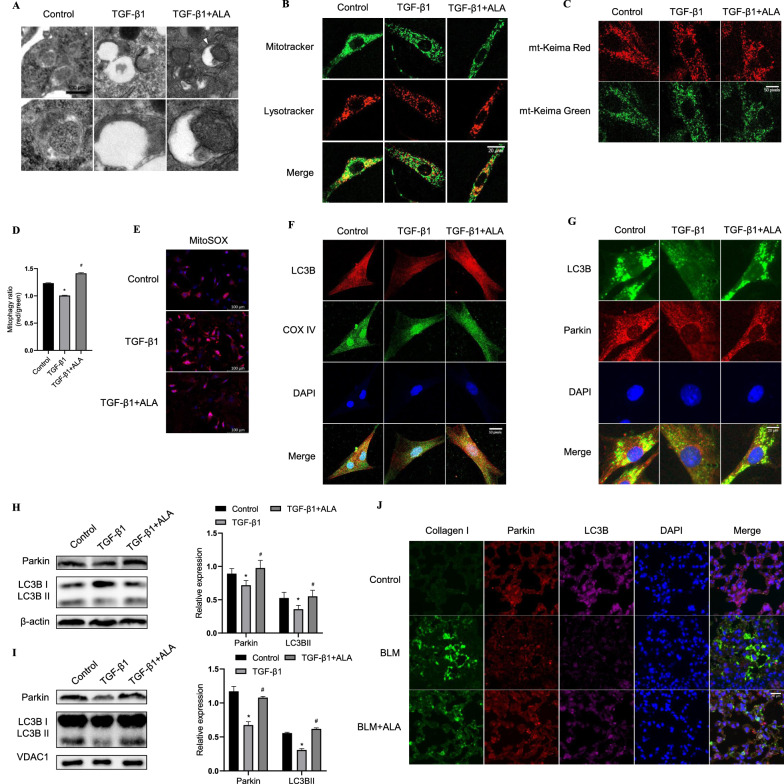
ALA upregulated Parkin/LC3B-driven mitophagy in lung fibroblasts. **A** Transmission electron microscopy analysis showing autophagosome/autolysosome (arrows) after treatment of lung fibroblasts. Bar: 500 μm. **B** Immunofluorescence analysis of the co-localization of Mitotracker and Lysotracker. Original magnification: 20 μm. **C** Mitophagy as determined by mt-Keima transfection. Green indicates mitochondria located in the cytoplasm, and red indicates mitochondria located in lysosomes. Original magnification: 50 pixels. **D** Mitophagy ratios are calculated as the ratio of red area to green area. **E** mtROS levels as examined using the mitoSOX indicator. Original magnification: 100 μm. **F**, **G** Co-localization of LC3B and COX IV or Parkin was assessed by confocal microscopy. Original magnification: 50 pixels and 20 μm. **H**, **I** Parkin and LC3B in cell lysate and mitochondrial components. **J** Co-localization of LC3B, Parkin, and collagen I in lung tissues. Original magnification: 20 μm. Data are presented as mean ± SEM. *P < 0.05 versus control group; ^#^P < 0.05 versus TGF-β1 group (n = 3)

Next, to determine whether metabolic reprogramming is related to mitochondrial homeostasis, we detected mitophagy levels and investigated how ALA regulated mitophagy in fibroblasts. After TGF-β1 treatment, mitochondria degradation by lysosomes declined in LF, while ALA addition visibly improved mitochondria degradation (Fig. 5A). Similarly, ALA increased the co-localization of Mitotracker and Lysotracker, which had been decreased by TGF-β1 (Fig. 5B). To further confirm the effect of TGF-β1 and ALA on mitophagic flux, cells were transfected with mt-Keima. Twenty-four hours after TGF-β1 treatment, the ratio of red to green areas had decreased, while ALA increased mitophagic flux (Fig. 5C, D). The MitoSOX assay also showed that levels of mitochondria reactive oxygen species (mitoROS) were reduced by ALA (Fig. 5E). These results explain how ALA could induce mitophagy to clear damaged mitochondria. Next, the co-localization of LC3B and COX IV (a mitochondrial marker) or Parkin was shown to be decreased by TGF-β1, but both co-localizations were further reversed by adding ALA, revealing that Parkin/LC3B-mediated insufficient mitophagy participated in the process of TGF-β1-induced LF activation and that ALA could upregulate mitophagy (Fig. 5F, G). The proteins expressed in cell lysate and mitochondria showed the same tendency (Fig. 5H, I). In vivo, ALA also upregulated the co-localization of Parkin and LC3B and fibroblast marker collagen I in lung tissue compared with that in the BLM group (Fig. 5J). Collectively, these data indicate that ALA could upregulate Parkin/LC3B-mediated mitophagy to repress TGF-β1-induced LF activation and BLM-induced PF.

### ALA upregulated mitophagy to repress fibroblast activation

**Fig. 6:**
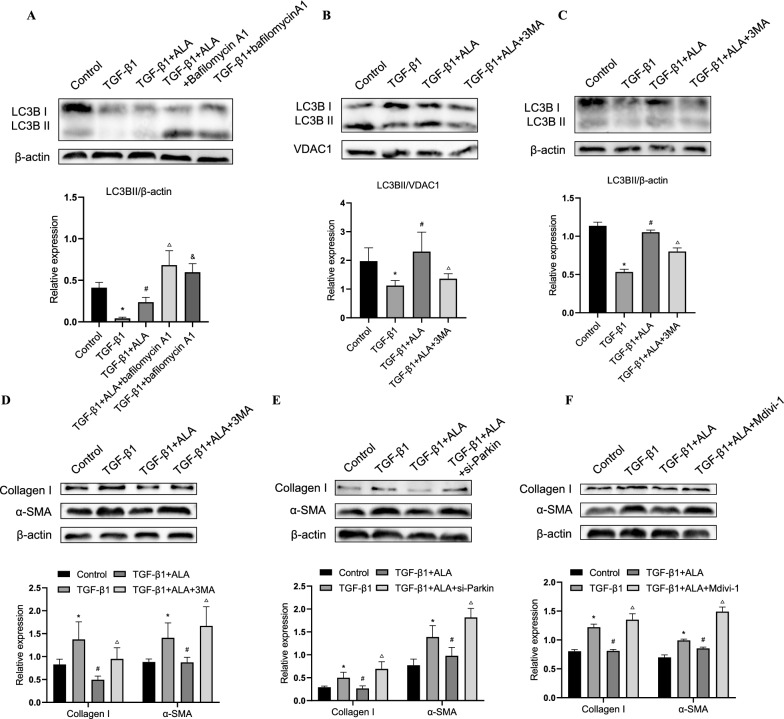
ALA upregulated Parkin/LC3B-mediated mitophagy to repress lung fibroblast activation. **A**–**C** Protein levels of LC3BII in cell lysate and mitochondrial components. **D**–**F** Collagen I and α-SMA were assessed after treatment with 3MA (10 μmol/L), parkin siRNA, or Mdivi-1 (10 μmol/L). Data are presented as mean ± SEM. *P < 0.05 versus control group; ^#^P < 0.05 versus TGF-β1 group; ^△^P < 0.05 versus TGFβ1 + ALA group; ^&^P < 0.05 versus TGFβ1 + ALA + bafilomycin A1 group (n = 3)

To determine why mitophagy was reduced by TGF-β1 and increased by ALA, we focused on LC3B. To clarify whether the increase in LC3B caused by ALA was a result of activated autophagy or blocked autophagic flux, cells were pretreated with bafilomycin A1, which blocks the fusion of autophagosomes and lysosomes. As shown in Fig. 6A, bafilomycin A1 further increased the expression of LC3BII, suggesting that the elevation of LC3BII levels by ALA was due to autophagy induction. We also found that inhibiting autophagy with 3MA could reverse the upregulation of LC3BII by ALA in both mitochondria components and cell lysate (Fig. 6B, C). 3MA could also mediate the expression of collagen I and α-SMA in fibroblast, suggesting that upregulation of LC3B by ALA may make up for the mitochondrial flux and therefore repress fibroblast activation (Fig. 6D). Next, we treated fibroblasts with Parkin siRNA or Mdivi-1 to uncover the relationship between mitophagy and fibroblast activation. Both reversed the inhibitory effect of ALA on collagen I and α-SMA (Fig. 6E, F). Therefore, we conclude that ALA may inhibit LF activation via the upregulation of Parkin/LC3B-mediated mitophagy.

### ALA prevented glycolysis via promoting mitophagy/autophagy of fibroblasts

**Fig. 7:**
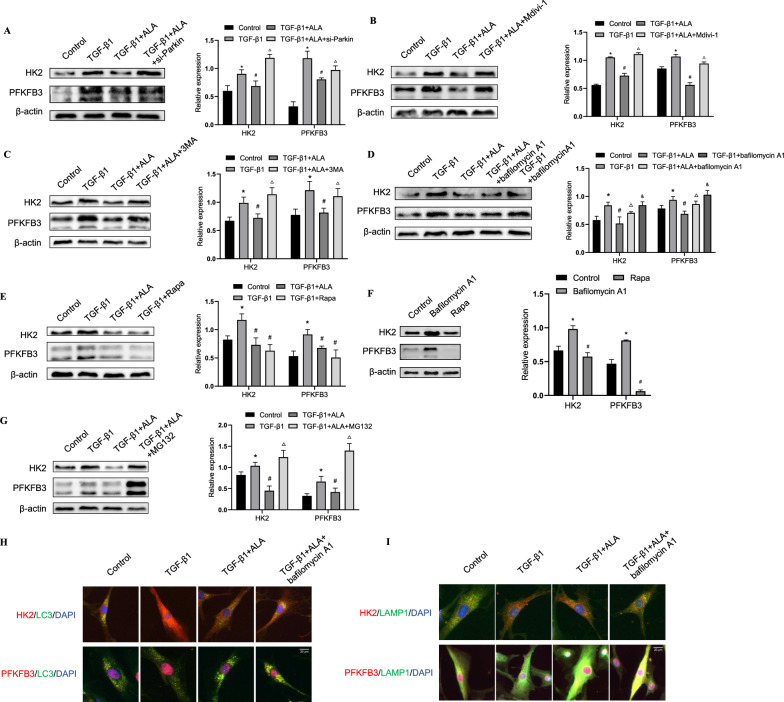
ALA prevented glycolysis by promoting mitophagy/autophagy in lung fibroblasts. **A**–**G** PFKFB3 and HK2 in cell lysate were measured after treatment with Parkin siRNA, Mdivi-1, 3MA, bafilomycin A1, Rapa, and MG132. **H**, **I** Co-localization of LC3 or LAMP1 and HK2 or PFKFB3, measured by immunofluorescence. Bar: 20 μm. Data are presented as mean ± SEM. *P < 0.05 versus control group; ^#^P < 0.05 versus TGF-β1 group; ^△^P < 0.05 versus TGFβ1 + ALA group; ^&^P < 0.05 versus TGFβ1 + ALA + bafilomycin A1 (n = 3)

Finally, to confirm the extent to which glycolysis was influenced by mitophagy, we evaluated the expression of PFKFB3 and HK2 in fibroblasts. The results showed that repressing mitophagy using a Parkin inhibitor or siRNA could reverse the suppression of PFKFB3 and HK2 by ALA (Fig. 7A, B). Moreover, 3MA and bafilomycin A1 upregulated the expression of HK2 and PFKFB3, but activation of autophagy with Rapa had the opposite effect (Fig. 7C–F). The co-localization of lysosome marker LAMP1 or autophagosome marker LC3 and HK2 or PFKFB3 is also upregulated by ALA, respectively (Fig. 7H, I). These results show that ALA could induce the autophagic degradation of HK2 and PFKFB3, which is repressed by TGF-β1. As HK2 and PFKFB3 could also be degraded by proteasomes, we investigated whether ALA represses their expression by inducing the proteasomes degradation pathway (Fig. 7G). Collectively, these results suggest that ALA inhibits TGF-β1-induced LF activation by repressing glycolysis via the promotion of mitophagy in fibroblasts.

### Bioinformatic analysis of glycolysis and mitophagy in LF and lung tissue isolated from IPF patients

Finally, we assessed the gene expression profiles of glycolysis and mitophagy in LF and lung tissue isolated from IPF patients by using the transcriptome data from GEO datasets (Additional file 1: Figs. S1, S2). In LF, the mRNA expression of COL1A1, a marker of activated fibroblasts, is clearly upregulated, while LC3B and PRKN, two mitophagy molecules, are obviously downregulated. In lung tissues isolated from IPF patients, the mRNA expression levels of COL1A1 and ACTA2, markers of activated fibroblasts, are upregulated, while PRKN is mildly downregulated. Two key glycolysis enzymes, HK2 and PFKFB3, show a small increase in fibroblasts and lung tissue, which is consistent with our conclusion.

## Discussion

**Fig. 8:**
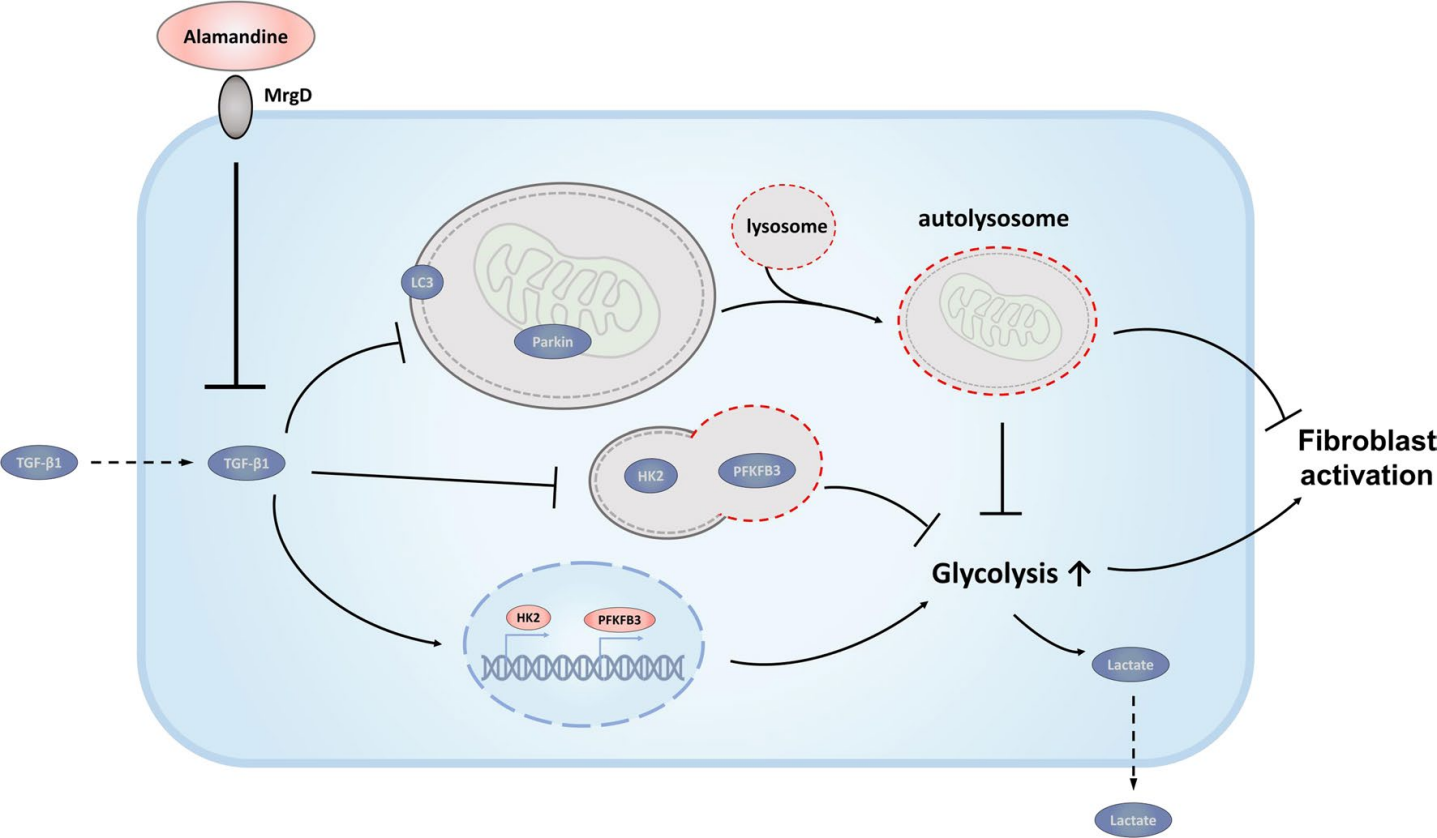
Schematic of proposed pathway depicting the role of ALA/MrgD receptor axis in fibroblasts

In the present study, we unraveled the mechanism of how the ALA/MrgD axis regulates LF activation in lung fibrosis. Our results showed that TGF-β1-induced LF activation was due to the regulation of metabolic reprogramming. TGF-β1 upregulated glycolysis by dysregulation of transcription levels of HK2 and PFKFB3 while inhibiting autophagic degradation of the corresponding proteins; both these effects were accompanied by a decrease in mitophagy, leading to a block in oxidative phosphorylation. All these effects were ameliorated by ALA (Fig. 8).

Studies have demonstrated that ALA plasmatic concentrations are decreased in IPF patients [24] and that ALA alleviates BLM-induced mice lung fibrosis and angiotensin II (Ang II)-induced fibroblast activation by binding to the MrgD receptor [16, 19]. Other studies found that myocardial, vascular, and liver fibrosis could be inhibited by ALA [17, 18, 25, 26]. However, the role of the ALA/MrgD axis in LF activation remained incompletely understood. Furthermore, interference with glycolysis switch PFKFB3 was found to rescue a mouse abdominal aortic aneurysm disease model induced by Ang II [27]. High glucose is closely related to aberrant glycolysis induction, and Ang II can increase glucose uptake, whereas angiotensin 1–7 (Ang 1–7) decreases it [28, 29]. Given this complex interplay and close associations with glucose metabolism of various components of RAAS, we explored how ALA regulated glycometabolism in LF by binding to the MrgD receptor.

Aerobic glycolysis, also called the Warburg effect, occurs when cells rely mainly on glycolysis to metabolize even in the presence of oxygen [30]. Glycolysis promotes fibroblast activation and proliferation and participates in the development of fibrosis in various organs, including the lung [3, 4, 31, 32]. Our findings presented here are consistent with those of previous studies. To explore whether ALA regulated the development of lung fibrosis through glycolysis, we detected the two main glycolytic switches and found that ALA could modulate the expression of PFKFB3 and HK2 at both the protein and transcript levels and induce them to be degraded by the autophagy-lysosome pathway. In line with Kottmann et al., we further found that lactate, the primary by-product of glycolysis, alone could induce fibroblast activation [22] while maintaining high expression of HK2 and PFKFB3. This indicates the existence of positive feedback involving glycolysis that maintains fibroblasts in their activated state, further supporting the continuous development of LF activation under inhibition by the ALA/MrgD receptor axis. Based on the finding that TGF-β1 activation is often accompanied by extremes of pH in vitro, we speculate that ALA represses lactate-induced fibroblast activation by inhibiting the activation of TGF-β1 [22, 33]; however, further study is needed to explore this in the future. Overexpression of PFKFB3 alone also induced fibroblast activation, suggesting that PFKFB3/lactate-driven glycolysis may have a pivotal role in maintaining fibroblast activation as well as being an indicator of activated fibroblasts. Furthermore, ALA increased the production of ATP repressed by TGF-β1, although the specific mechanism by which this occurred was not explored in the current work. As ALA has many similarities in terms of structure and function with Ang 1–7, we speculate that ALA may increase ATP production by upregulating the expression of citrate synthase, a key enzyme in the tricarboxylic acid cycle, further accelerating the oxidative phosphorylation process to promote ATP production in the same way as Ang 1–7 [34]. The evidence suggests that ALA may be a promising therapeutic target for the repression of LF activation through targeted glycolysis pathways.

Mitophagy is a crucial mean of mitochondrial quality control [10, 35–37]. The co-localization of LC3 and mitochondria indicates that mitophagy is in progress [38]. Reduced expression of Parkin and LC3B, two of the crucial factors in this process, indicates insufficient mitophagy in activated fibroblasts [39, 40]. The expression of Parkin in fibroblasts has been shown to decline after treatment with TGF-β1 [41]. Here, we observed reduced expression of Parkin and LC3B both in cell lysate and mitochondria after treatment with TGF-β1. ALA upregulated the production of LC3B to induce autophagy and repressing autophagy with 3MA abolished the anti-fibroblast-activation effect of ALA. We speculated that the upregulation of LC3B by ALA compensated for the mitochondrial flux, thereby maintaining the nonactivated state of fibroblasts. MitoROS, an indicator of mitochondrial homeostasis, was upregulated by TGF-β1 but downregulated by ALA, similar to the effects of Ang 1–7 reported in our previous work [42]; this indicates that insufficient mitophagy flux may cause large numbers of abnormal mitochondria to accumulate in cells such that mitoROS is not removed effectively. Moreover, Li Xiao et al*.* found that mitoQ, a mitochondria-targeted antioxidant that removes superfluous mitoROS, could also upregulate mitophagy by promoting Parkin expression. All these results indicate a close relationship between mitoQ and mitophagy. How mitoROS affect glycolysis and fibroblast activation through improving mitophagy will be studied in more detail in future research.

As mitochondria are the pivotal organelles for glucose metabolism, our results indicate that abnormal mitophagy may be the main reason for the enhanced glycolysis observed in lung fibrosis. The role of mitophagy in metabolic reprogramming has been controversial [43, 44]. Mitophagy-dependent loss of mitochondria has been reported to induce glycolysis during tumor cells' mitotic arrest [39]. Mitophagy deficiency was also found to lead to aerobic glycolysis, whereas restoration of Parkin could reverse this effect [45]. However, the specific relationship between mitophagy and glycolysis has not been elucidated, nor has the mechanism by which they regulate fibroblast activation and lung fibrosis. In the present study, downregulating Parkin or blocking autophagy flux with bafilomycin A1 increased the expression of glycolysis key molecules; therefore, we concluded that Parkin/LC3B-driven mitophagy/autophagy influences the autophagic degradation of HK2 and PFKFB3 in LF, with further repression of fibroblast activation by ALA through activating mitophagy/autophagy. Zhang et al. previously showed that mitophagy and glycolysis are not regulated in one direction and that glycolysis could also regulate mitophagy through different targets [46]; however, further studies are needed to determine whether the latter leads to LF activation. We also analyzed the public databases from NCBI and found that compared to the normal control group, the tendency of glycolysis and mitophagy in LF and lung tissue isolated from IPF patients are similar to our conclusions. Other researches have also found that the expressions of glycolysis increased, while mitophagy-related molecules LC3B and Parkin decreased in the lung tissue of IPF patients [3, 4, 39, 47]. Despite the promising implications of the finding that ALA alleviated fibroblast pro-fibrotic activity, the present study was limited in that it did not further evaluate the expression of ALA or the relationship between glycolysis and mitophagy in patients with IPF. These aspects will be examined using further experiments in future studies.

## Conclusion

In conclusion, we present a previously unrecognized mechanism by which the ALA/MrgD axis could inhibit TGF-β1-induced LF activation by counteracting glycolysis via activating Parkin/LC3-mediated mitophagy. This indicates a potential new strategy for targeted therapy of lung fibrosis with potential clinical applications.

## Supplementary Information

**Additional file 1: Figure S1**. Gene expression of glycolysis and mitophagy in fibroblast isolated from IPF patients. **A** This heatmap shows the expression level of glycolysis-related and mitophagy-related genes. **B** Raw counts were converted into transcripts per million (TPM) for comparison in different samples. Boxplot of transcripts per million (TPM) showing glycolysis-related, mitophagy-related, and marker genes in fibroblast that with slight variation and corresponding trends. **Figure S2**. Gene expression of glycolysis and mitophagy in lung tissue isolated from IPF patients. **A** This heatmap shows the expression level of glycolysis-related and mitophagy-related genes. **B** Raw counts were converted into transcripts per million (TPM) for comparison in different samples. Boxplot of transcripts per million (TPM) showing glycolysis-related, mitophagy-related, and marker genes in lung tissue with slight variation and corresponding trends.

## Data Availability

All data generated or analyzed during this study are included in this published article.

## Abbreviations

IPF: Idiopathic pulmonary fibrosis
PF: Pulmonary fibrosis
LF: Lung fibroblast
ALA: Alamandine
MrgD: MAS-related G-protein coupled receptor D
ECM: Excessive extracellular matrix
BLM: Bleomycin
TGF-β1: Transforming growth factor-beta1
α-SMA: α-Smooth muscle actin
RAAS: Renin–angiotensin–aldosterone system
HK2: Hexokinase 2
PFKFB3: 6-Phosphofructo-2-kinase/fructose-2,6-biphosphatase 3
COX IV: Cytochrome c oxidase IV
LC3B: Light chain 3B
3MA: 3-Methyladenine
H&E: Hematoxylin and eosin
qRT-PCR: Quantitative real-time polymerase chain reaction
