# Supplementary material for: Alamandine/MrgD axis prevents TGF-β1-mediated fibroblast activation via regulation of aerobic glycolysis and mitophagy
Source: J Transl Med. 2023 Jan 13;21:24. doi: 10.1186/s12967-022-03837-2 (PMC9838062; doi:10.1186/s12967-022-03837-2)

Figure S1. Gene expression of glycolysis and mitophagy in fibroblast isolated from IPF patients. **(A)** This heatmap shows the expression level of glycolysis-related and mitophagy-related genes. **(B)** Raw counts were converted into transcripts per million (TPM) for comparison in different samples. Boxplot of transcripts per million (TPM) showing glycolysis-related, mitophagy-related and marker genes in fibroblast with slight variation and corresponding trends.

A

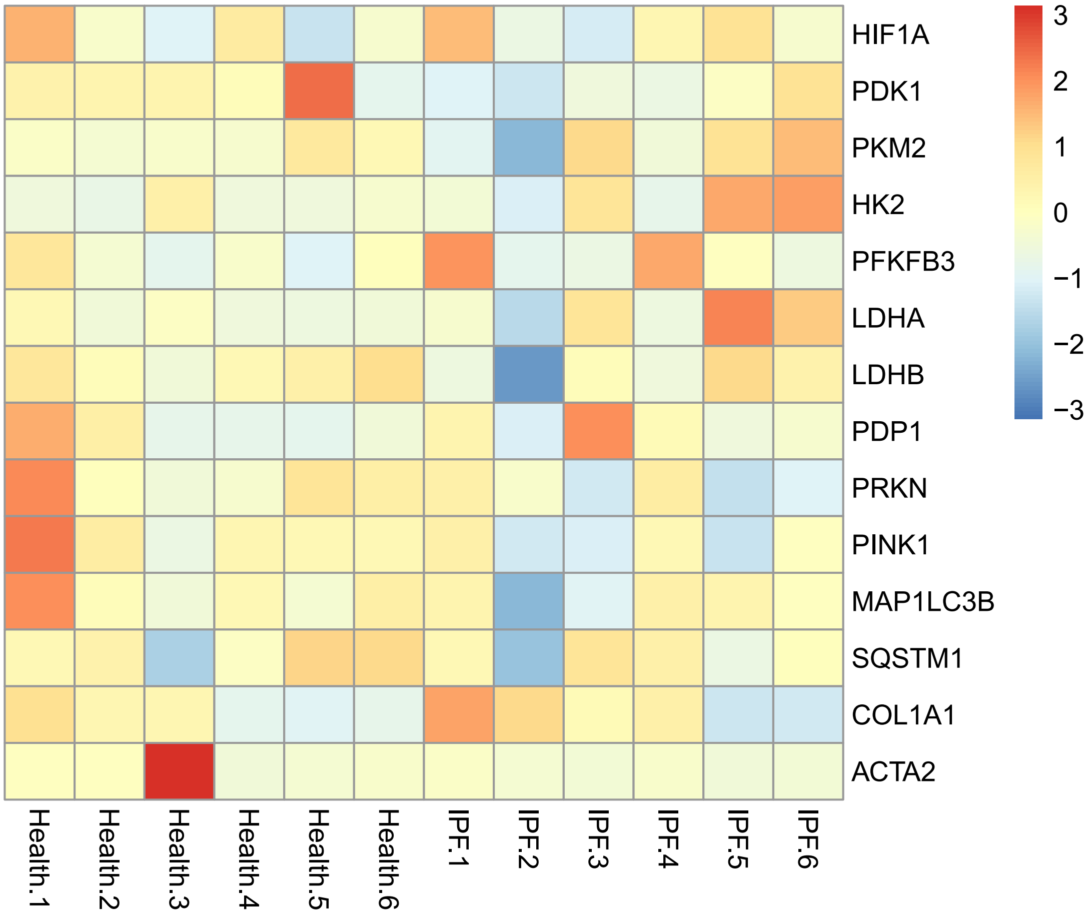

B

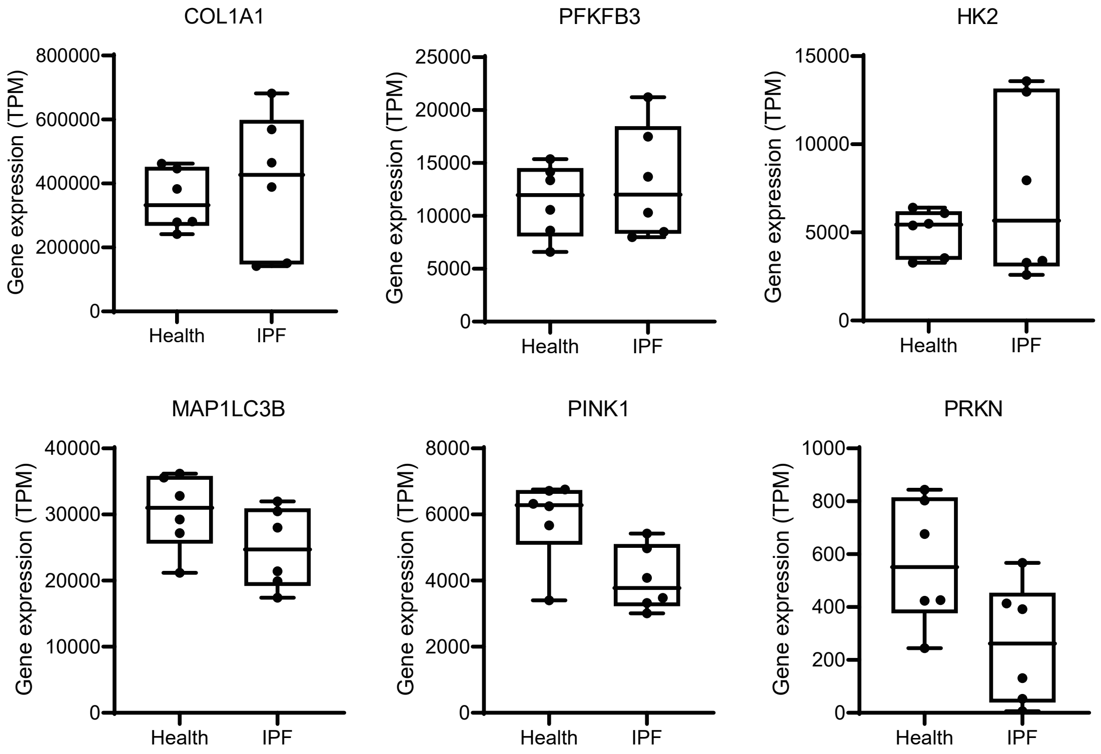

Figure S2 Gene expression of glycolysis and mitophagy in lung tissue isolated from IPF patients. **A** This heatmap shows the expression level of glycolysis related and mitophagy related genes. **B** Raw counts were converted into transcripts per million (TPM) for comparison in different samples. Boxplot of transcripts per million (TPM) showing glycolysis related, mitophagy related and marker gene in lung tissue that has slight variation and corresponding trends.

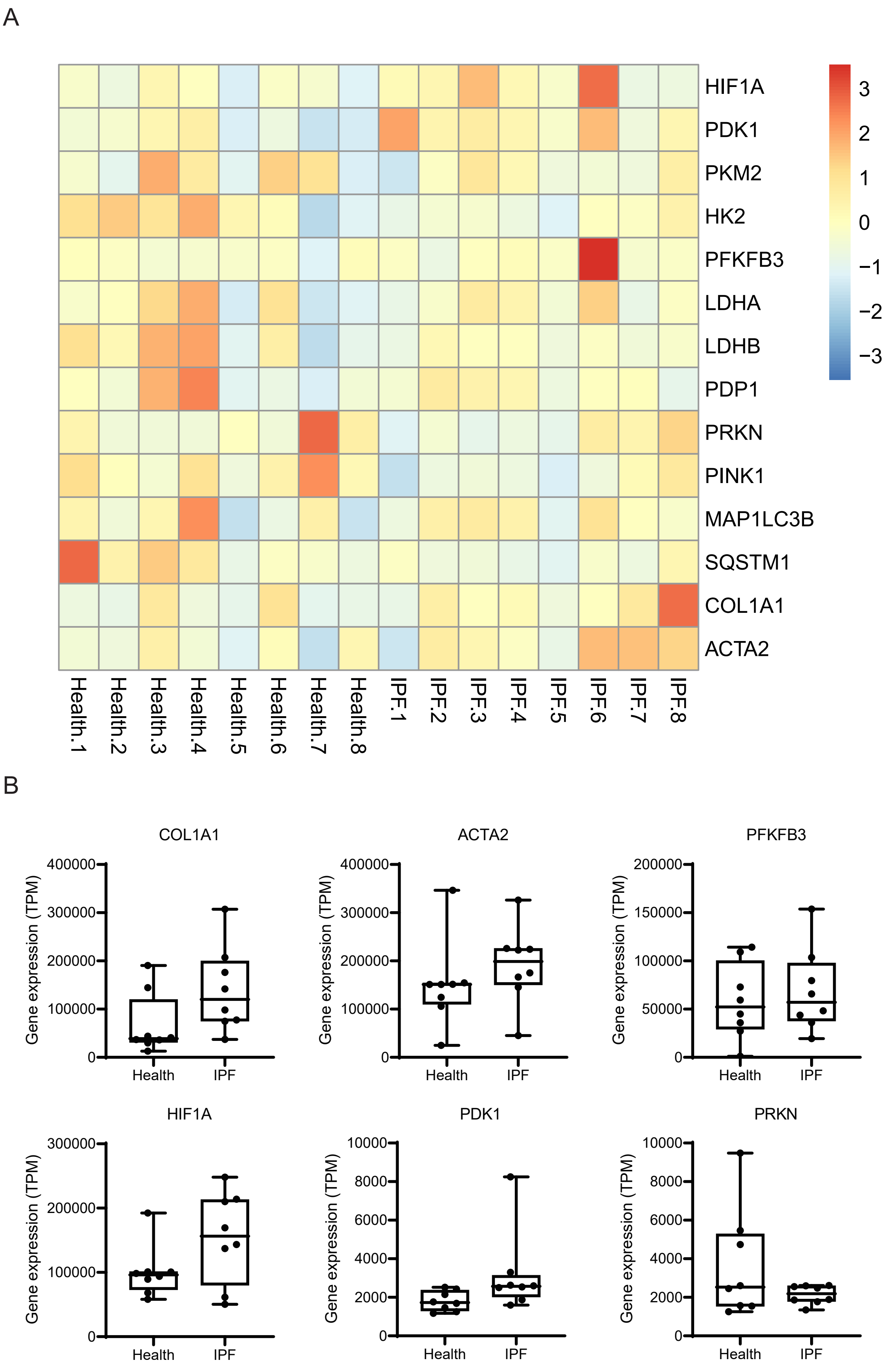

Supplement: Supplementary file 1 — Additional file 1: Figure S1. Gene expression of glycolysis and mitophagy in fibroblast isolated from IPF patients. A This heatmap shows the expression level of glycolysis-related and mitophagy-related genes. B Raw counts were converted into transcripts per million (TPM) for comparison in different samples. Boxplot of transcripts per million (TPM) showing glycolysis-related, mitophagy-related, and marker genes in fibroblast that with slight variation and corresponding trends. Figure S2. Gene expression of glycolysis and mitophagy in lung tissue isolated from IPF patients. A This heatmap shows the expression level of glycolysis-related and mitophagy-related genes. B Raw counts were converted into transcripts per million (TPM) for comparison in different samples. Boxplot of transcripts per million (TPM) showing glycolysis-related, mitophagy-related, and marker genes in lung tissue with slight variation and corresponding trends. [file 12967_2022_3837_MOESM1_ESM.pdf]
